# A tiered approach to genome-wide association analysis for the adherence of hulls to the caryopsis of barley seeds reveals footprints of selection

**DOI:** 10.1186/s12870-019-1694-1

**Published:** 2019-03-06

**Authors:** Celestine Wabila, Kerstin Neumann, Benjamin Kilian, Volodymyr Radchuk, Andreas Graner

**Affiliations:** 1 Leibniz Institute of Plant Genetics and Crop Plant Research (IPK), 06466 Seeland, Germany; 2 Present address: Global Crop Diversity Trust, Platz der Vereinten Nationen 7, 53113 Bonn, Germany; 3 Martin-Luther-University Halle-Wittenberg, Betty-Heimann-Str. 3, 06120 Halle/Saale, Germany

**Keywords:** GWAS, Naked caryopsis, *Nud*, Domestication, Seed protein content

## Abstract

**Background:**

Seeds of domesticated barley are grouped into two distinct types, which differ in morphology. Caryopses covered by adaxial (*palea*) and abaxial (*lemma*) hulls that tightly adhere to the pericarp at maturity give rise to hulled seeds whereas caryopses without adhering hulls give rise to naked seeds. The naked caryopsis character is an essential trait regarding the end use of barley.

**Results:**

To uncover the genetic basis of the trait, a genome-wide association study (GWAS) has been performed in a panel comprising 222 2-rowed and 303 6-rowed spring barley landrace accessions. In addition to the well-described *Nud* locus on chromosome 7H, three novel loci showed strong associations with the trait: the first locus on 2H was specifically detected in 6-rowed accessions, the second locus on 3H was found in 2-rowed accessions from Eurasia and the third locus on 6H was revealed in 6-rowed accessions from Ethiopia. PCR analysis of naked accessions also confirmed the absence of a 17 kb region harboring the *Nud* gene on chromosome 7H for all but one naked accession. The latter was characterized by a slightly variant phenotype of the caryopsis.

**Conclusion:**

Our findings provide evidence of the pervasiveness of the 17 kb deletion in spring barley from different geographic regions and at the same time reveal genomic footprints of selection in naked barley, which follow both geographic and morphological patterns.

**Electronic supplementary material:**

The online version of this article (10.1186/s12870-019-1694-1) contains supplementary material, which is available to authorized users.

## Background

Barley (*Hordeum vulgare* L. ssp. *vulgare*) ranks among the early founder crops of the Fertile Crescent [1]. Contrary to early reports pointing to a monophyletic origin of domesticated barley within the Levant [2], recent reports assign a much wider region of barley domestication in West and East Asia with multiple centers of domestication [3–5]. In order to study the domestication history of barley, several traits related to the domestication syndrome have been investigated and the corresponding genes isolated, e.g. brittleness of the rachis [6], spike morphology such as row type [7–12] and morphotype of the caryopsis [13]. The spikes of wild barley (*Hordeum vulgare* L. ssp. *spontaneum*) are characterized by hulled caryopses. Here, seeds are enclosed by two firmly adhering hulls, the palea on the ventral and the lemma on the dorsal side. These protect the embryo from mechanical damage during seed dispersal and in the soil.

Hulless or naked seeds occurred already early in the domestication of barley and can be traced back to archaeological finds at Ali Kosh from c. 9000 years ago [14, 15]. The described variation in seed morphotype is of agronomic relevance because it is directly linked to the end use of barley. Particularly in Asia and many parts of Africa including Ethiopia, naked barley represents an important food source [16]. Covered barley seeds, on the other hand, are mainly used as animal feed and for beer production. Regarding the latter, hulls provide a filtration medium when processing the wort for fermentation [17]. Harlan [18] reported a sticky, adhesive substance that appears 10 days after flowering on the surface of the developing pericarp tissue of hulled barley seeds. This substance has been identified as a cuticular lipid, whose presence or absence on the epidermal layer of the pericarp regulates the adherence of the hulls giving rise to covered or naked seed [13, 19]. The covered/naked caryopsis in barley is controlled by a single locus (*nud*, for *nudum*) located on the long arm of barley chromosome 7H [20]. The corresponding gene encodes an ethylene response factor *(ERF)* family transcription factor which is involved in lipid biosynthesis [13].

Although to date no locus other than *Nud* has been reported for the naked caryopsis, both the complex biosynthesis of lipids as well as the vast genetic diversity of barley suggest that naked caryopses seen in barley landraces from different geographic regions may have arisen from allelic variation in more than only one gene.

To test this hypothesis, we carried out a genome-wide association analysis (GWAS) on a diverse collection consisting of 222 2-rowed and 303 6-rowed spring barley landraces, which were characterized by the presence/absence of a hulled caryopsis. To minimize the effect of population structure, we performed GWAS separately for the two subpopulations of 2- and 6-rowed accessions and further refined our approach by individually analyzing naked genotypes of either Ethiopian or European/Asian (Eurasian) origin. As a result, we confirmed (i) that a characteristic deletion of a chromosomal fragment including the *Nud* gene was pervasive in our panel consisting of naked barleys from a wide range of geographical regions, and (ii) that this deletion is accompanied by strong footprints of selection mirrored by allele frequencies that correlate with geographical patterns and spike morphology. In addition, we identified a single naked accession (HOR1143) originating from Ethiopia which does not carry the diagnostic deletion and which was characterized by an intermediate phenotype*.*

## Methods

### Plant material

The association panel consisted of 222 2-rowed and 303 6-rowed spring barley landraces (*Lr*) selected from a core set described before [21]. The barley accessions can be traced to 35 countries and the following geographical regions: North and East Africa (*n* = 155), Southern Europe (*n* = 100), North and Central Europe (*n* = 38), Eastern Europe (*n* = 138), Near and Middle East (94). All accessions were geo-referenced ranging from 16.6° western to 71.5° eastern longitude and from 5.8° to 61.9° northern latitude (Additional files 1 and 2). The 2-rowed panel comprised 178 hulled accessions, 28 naked accessions from Ethiopia and 16 naked accessions from Eurasia, while the 6-rowed panel consisted of 257 hulled accessions, 28 naked genotypes from Ethiopia and 18 naked genotypes from Eurasia (Additional file 3: Figure S1). All accessions were obtained from the Federal ex situ Genebank at IPK (Gatersleben, Germany) and purified over several rounds of single seed descent to ensure their homozygous state. For association analysis, the barley landrace population (*Lr_all*) was split into the following subpopulations: (i) 2-rowed lines (*Lr_2*), (ii) 6-rowed lines (*Lr_6*), (iii) 6-rowed lines with naked accessions originating only from Ethiopia (*Lr_6Eth*) or (iv) from Eurasia (*Lr_6Eur*) and (v) 2-rowed lines including naked barley only from Ethiopia (*Lr_2Eth*) or (vi) from Eurasia (*Lr_2Eur*). Geographic origin of all accessions used in the current study is presented as supplementary information (Additional file 3: Figure S2).

### Phenotyping

**Fig. 1:**
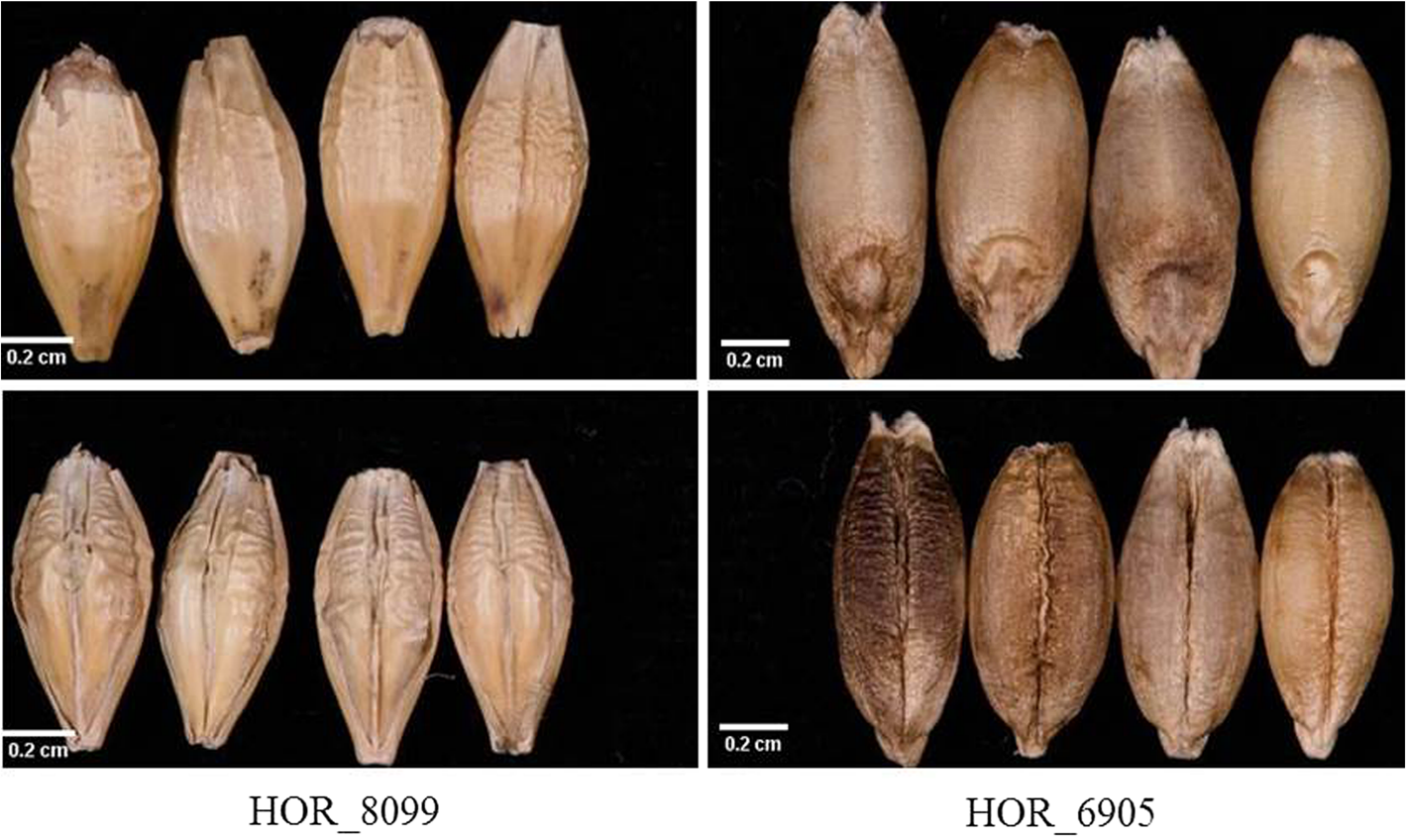
Barley seeds. Top panel: dorsal side of hulled seeds (left) and naked seeds (right); lower panel; ventral side of hulled seeds (left) and naked seeds (right)

Seeds of individual accessions were scrutinized under a binocular microscope at × 25 magnification to validate the passport data on the seed type obtained from the Genebank. For GWAS, hulled accessions were given a score of 1, while naked accessions were given a score of 2 (Fig. 1).

### Genotyping

Analysis of single nucleotide polymorphisms (SNPs) was performed using the barley iSelect 9 k chip (Illumina, San Diego, USA) with SNP selection criteria described in [22]. About 5 g of fresh leaf material from two weeks old seedlings was harvested, shock-frozen in liquid nitrogen and stored at − 80 °C until DNA extraction. DNA was extracted according to the Cetyltrimethyl Ammonium Bromide (CTAB) DNA Miniprep protocol [23]. SNP analysis was performed by a service provider (Trait Genetics GmbH, Gatersleben, Germany). The iSelect array comprises 7864 SNPs. Genetic positions of SNPs were determined by reference to the POPSEQ genetic map [24] and the Morex x Barke genetic map [22]. In total, 5885 SNPs were represented on at least one map. From these, 5312 polymorphic SNPs were present in the entire panel of 2- and 6-rowed accessions while in the 2-rowed and 6-rowed sub panels 4971 SNPs and 4965 SNPs were detected, respectively. After excluding SNPs with a minor allele frequency (MAF) below 5% and SNPs with more than 5% missing data, 4791 polymorphic SNPs were finally considered for GWAS of the entire population. The 2-rowed and the 6-rowed subpanels comprised 4719 and 4349 polymorphic SNPs, respectively.

### Population structure and LD decay

Principal component analysis (PCA) was performed employing the software package R 2.15.3 [25] [26]. The extent of average genome-wide Linkage disequilibrium (LD) decay was computed for individual GWAS panels by using the software package GenStat 16th edition [27]. Pair-wise marker correlation *r*^*2*^ for individual chromosomes was calculated and plotted against the map distance, and a critical threshold value was derived from the 95th percentile of the distribution of the *r*^*2*^ values from all unlinked (> 50 cM) loci according to Breseghello and Sorrells [28]. Beyond this value, LD was assumed to be caused by genetic linkage. A Loess curve was then fitted to all *r*^*2*^ values, and the point of interception between the Loess curve and the *r*^*2*^ threshold value was taken as an estimate for the average genome-wide LD decay.

LD as a function of *r*^*2*^ between significant SNPs was visualized by heat plots generated in the software package Haploview 4.2 [29]. For this, the default settings of 500 kb between SNPs was replaced with zero to force all pairwise computations since the physical map distance was not relevant in our calculation.

### Association analysis and calculation of false discovery rate

All association analyses were calculated by running GAPIT package in R 2.15.3 [26] as described by [30]. GWAS was conducted by applying the compressed mixed linear model (CMLM), which includes both fixed and random terms. By including individuals as random effects, the CMLM incorporates information about relationships between individuals. We jointly used information from a genetic marker-based kinship matrix (K) and population structure calculated by PCA in the CMLM approach improving the statistical power of the GWAS while controlling for spurious associations. The proportion of the total variance explained by the genetic variance is defined as heritability (*h*^*2*^) with σ^2^_a_ as the additive genetic and σ^2^_e_ the residual variance.

$$ {h}^2={\sigma^2}_a/\left({\sigma^2}_a+{\sigma^2}_e\right) $$

Cluster analysis was used to assign individuals into groups with the elements of the kinship matrix used as similarity measures in the cluster analysis. The number of groups used in the calculation was PCA = 5.

Manhattan plots of GWAS results were generated using “CMplot” by running the “CMplot” package in R 3.3.1. We implemented the false discovery rate (FDR) of 5% [31] to account for multiple testing and to determine a threshold for true positive associations.

### PCR amplification and resequencing

To investigate the presence of a characteristic deletion spanning the *Nud* locus in naked barleys, we performed PCR amplification using a combination of three primers, wF2, kR1, tR2, as described by [13]. Hulled barleys were included in the analysis as positive controls. According to [13], the PCR will detect the presence or absence of a 17-kb fragment by an amplicon length polymorphism (853 bp in hulled and 785 bp in naked barley, respectively). Primer sequences and PCR conditions are described in the supplementary section (Additional file 3: Table S3). We sequenced amplicons from all naked accessions from both ends (using wF2 for the forward, tR2_reverse for covered and KR1_reverse for naked barley, respectively) using an established Sanger sequencing procedure (BigDye™ Terminator v3.1 Cycle Sequencing Kit, Applied Biosystems™ 3730xl DNA Analyzer. To confirm the extent of the deletion, sequences of all accessions were aligned to the reference sequence of cv Morex, a 6-rowed cultivar with hulled caryopsis, using the sequence alignment software package Sequencher 5.2.4 (genecodes.com).

### Protein content analysis

The protein content of naked accessions was measured by applying a non-destructive near-infrared reflectance spectroscopy method according to the protocol of [32]. For this, five sets of 30 grains each were sampled from each accession. All seed samples used for analysis were harvested in the same year (2011, Gatersleben). The final protein content was averaged from all five technical replications.

## Results

### Population structure and LD decay

**Fig. 2:**
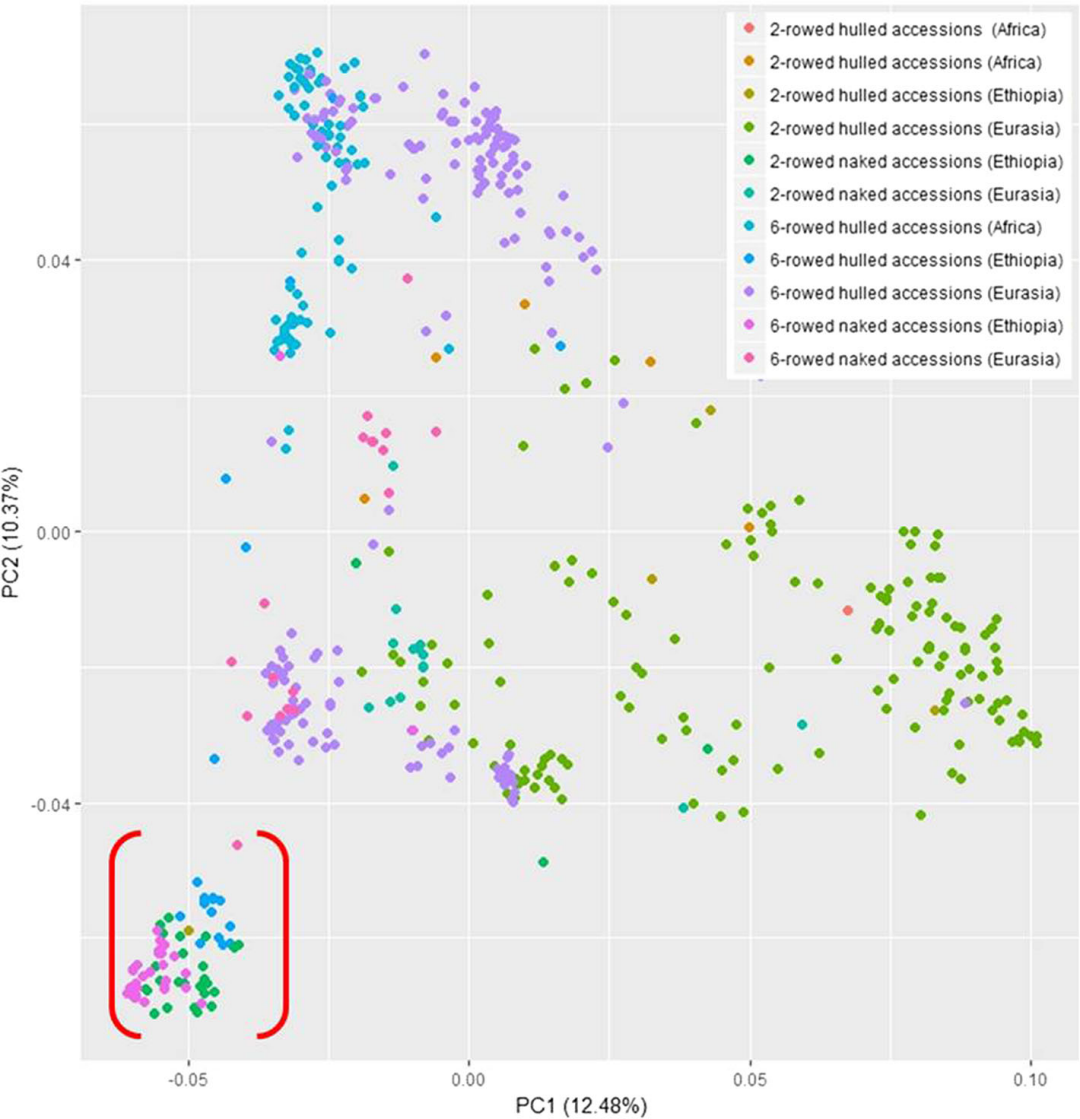
PCA for the entire *Lr_all* panel of 525 spring barley landrace accessions (both 2- and 6-rowed) based on iSelect SNP data. The unique grouping of accessions from Ethiopia (naked 2- and 6-rowed together with hulled 6-rowed accessions) is indicated by brackets

PCA for the whole GWAS panel *Lr_all*, including both 2- and 6-rowed accessions was performed to obtain information about genetic stratification within the population (Fig. 2). The first two PCs explained 12.48 and 10.37% of the total genotypic variance. Most accessions from Ethiopia grouped at a position distinct from the rest of the remaining accessions underscoring their unique genetic makeup and confirming previous findings of [21], which were based on SSR markers. Most of the genetic variance explained in PC1 could be attributed to the difference in row type (2-rowed vs 6-rowed), while most of the genetic variance explained in PC2 could be attributed to the geographical origin (Ethiopia vs the rest). Here, most of the 2-and 6-rowed Ethiopian barley (both naked and covered) grouped as indicated by red brackets on the PCA (Fig. 2).

For both panels, *Lr_2* and *Lr_6*, a significant drop in pairwise SNP correlation (LD) was observed. The decrease in LD was faster in the 6-rowed than in the 2-rowed panel as many SNPs located within a genetic distance of 10 cM in the 2-rowed panel were still in significant LD (*r*^*2*^= > 0.5) (Additional file 3: Figure S3). Average genome-wide LD decay was estimated at 3 cM and 2 cM in *Lr_2* and *Lr_6*, respectively. The LD decay observed in the present panel is in general accordance with results obtained in other barley landrace populations [33, 34].

### Association analysis

GWAS revealed a large number of significant marker-trait associations for the hulled/naked character. In the following, we will focus only on the subset of associations that exceeded the FDR threshold.

**Fig. 3:**
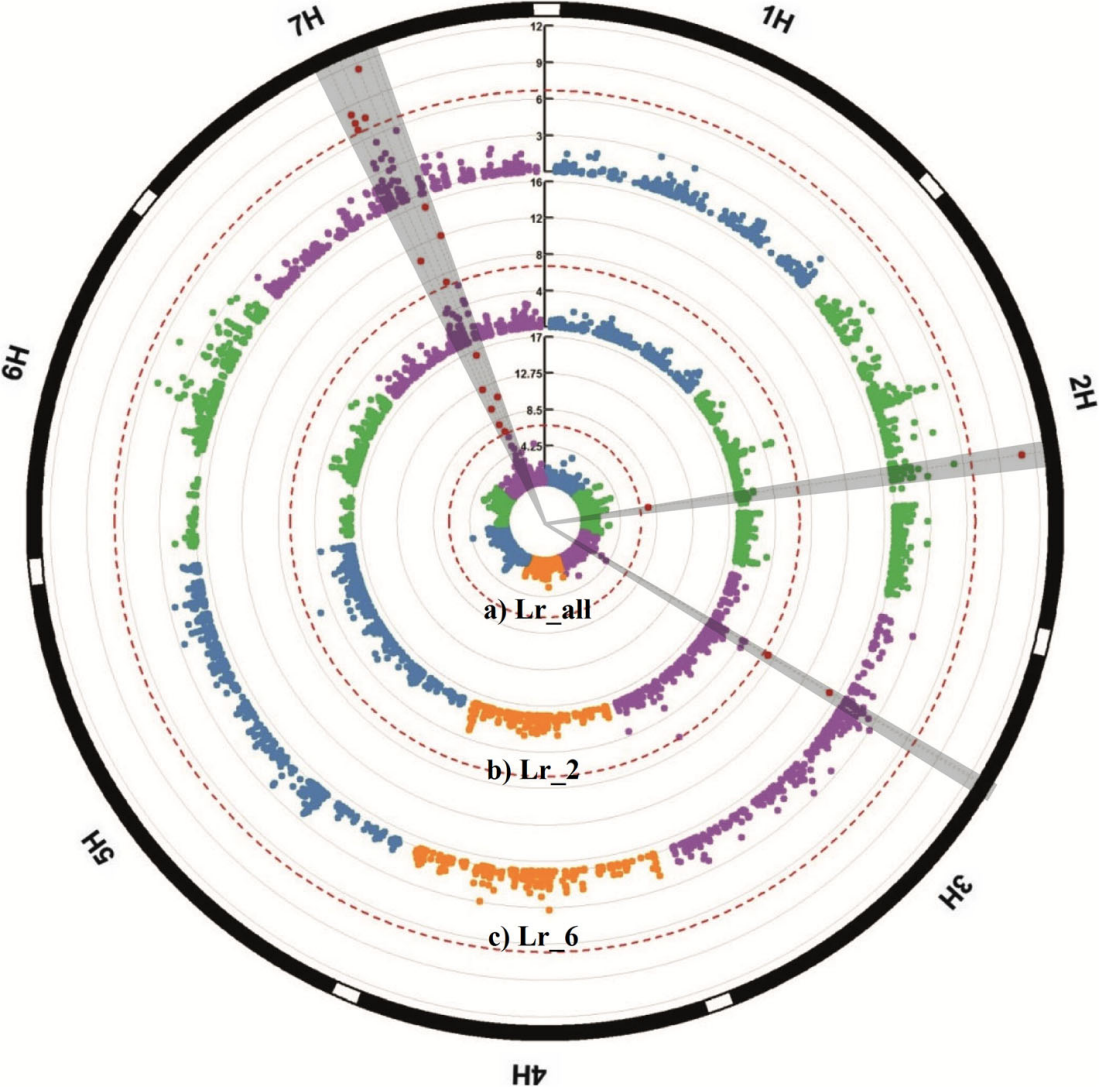
Circular Manhattan plots of genome-wide association scans for seed morphotype (covered/naked) in (**a**) the combined panel of 2- and 6-rowed barley landraces (*Lr_all*), **b**) the subpopulation of 2-rowed (*Lr_2*), and **c**) the subpopulation of 6-rowed accessions (*Lr_6*). The significance level of marker-trait associations (−logP values) is represented by the vertical scale bar. Individual chromosomes are represented on the outer circle and separated from each other by white borders. FDR thresholds (0.05) are indicated by dashed circles. Genomic regions of detected QTL on the respective chromosomes are coloured in red (outer circle). Owing to the limited resolution of the figure single dots can represent multiple SNPs, especially if they have the same or very similar -logP values

Analysis of *Lr_all* revealed seven SNPs on chromosome 7H within an interval of 13.6 cM (70.8 cM – 84.4 cM). An additional QTL was marked by a single SNP on the long arm of chromosome 2H (91.2 cM) (Fig. 3, *Lr_all*).

When restricting the analysis to *Lr_2*, eight SNPs detected two loci, one on the short arm of chromosome 3H and as in *Lr_all* on 7HL (Fig. 3, *Lr_2*). The 3HS locus is marked by four SNPs mapping at 45.4 cM (−log *P* = 7.2), 46 cM (2 SNPs each with –log *P* = 15.1 and one SNP with –logP = 7.2). The four SNPs on 7HL map to an interval of 15.2 cM at 70.8 cM (−logP = 10.3), 79.8 (−logP = 7.1), 84.4 cM (−logP = 15.6) and 86.6 cM (−logP = 12.1). The most significant SNP on 7HL at 84.4 cM explained about 21% of the total phenotypic variance, while the two most significant SNPs on 3H (46 cM) explained 17% of the total phenotypic variance (Additional file 3: Table S4). Regarding subpanel *Lr_6*, two major loci were detected. In accordance with the *Lr_2* and *Lr_all* panel, the 7HL locus was marked by six SNPs. The highest association on 7HL was detected at 79.8 cM (−logP = 11.5), explaining 14.9% of the total phenotypic variance. A second locus was identified on chromosome 2HL by a single SNP located at 91 cM (−logP = 11) explaining 14.7% of the total phenotypic variance (Fig. 3, Additional file 3: Table S4).

### Identification of subpopulation-specific associations

Out of 90 barley accessions with naked caryopses, 56 originate from Ethiopia. As Ethiopian barley landraces formed the largest and genetically most distinct group of naked barley in our mapping panel, we decided to scrutinize this group separately. To this end, GWAS was performed individually by including only naked barleys of Ethiopian or of non-Ethiopian (Eurasian) origin in the 2-rowed (*Lr_2Eth; Lr_2Eur*) and the 6-rowed sub-populations (*Lr_6Eth*; *Lr_6Eur*). The number of accessions in each panel is presented in Table 1. Again, significant associations on chromosome 7HL were detected in all subgroups. However, associations on the other chromosomes followed a geographic pattern (Additional file 3: Table S5).

**Table 1:** Summary of chromosomal associations detected for the phenotype naked caryopsis

Mapping panel	Chromosome					
	2H			3H	6H	7H
	58.8–58.9 cM	78.6 cM	91–91.2 cM	45.4–51.1 cM	65.9 cM	70.8–86.6 cM
*Lr_all (435 hulled, 90 naked)*			1 SNP (R^2^ = 10.2)			7 SNPs (R^2^ range: 9–13)
*Lr_2 (178 hulled, 44 naked)*				4 SNPs (R^2^ range:(14–17)		4 SNPs (R^2^ range: 16–21)
*Lr_2 Eth (178 hulled, 28 naked)*						4 SNPs (R^2^ range: 27–34)
*Lr_2 Eur (178 hulled, 16 naked)*				5 SNPs (R^2^ range: 18–24.4)		1 SNP (R^2^ = 24)
*Lr_6 (257 hulled, 46 naked)*			1 SNP (R^2^ = 14.7)			6 SNPs (R^2^ range: 12–14.9)
*Lr_6Eth (257 hulled, 28 naked)*	5 SNPs (R^2^ range: 13–15.5)	1 SNP (R^2^ = 13)			2 SNPs (R^2^ = 13)	1 SNP (R^2^ = 14)
*Lr_6Eur (257 hulled, 18 naked)*	2 SNPs (R^2^ = 13)		2 SNPs (R^2^ range: 13–15)			6 SNPs (R^2^ range: 12.7–17.8)
*2H 58.8–58.9 cM SNPs shared by panels*	BOPA2_12_11316, BOPA2_12_21476			*Lr_6Eth, Lr_6Eur*		
*2H 91–91.2 cM SNPs shared by panels*	SCRI_RS_140972			*Lr_all, Lr_6, Lr_6Eur*		
*3H 45.4–51.1 cM SNPs shared by panels*	BOPA1_2391–566, BOPA1_4256–833, BOPA2_12_10532, BOPA2_12_30474			*Lr_2, Lr_2Eur*		
*7H 70.8–86.6 cM SNPs shared by panels*	BOPA2_12_20685			*Lr_all*, *Lr_6*, *Lr_2*, *Lr_2Eur*, *Lr_6Eur*		
	BOPA2_12_20685, SCRI_RS_4562			*Lr_all*, *Lr_2Eth*, *Lr_6*, *Lr_6Eth*		
	BOPA1_1676–557, BOPA2_12_11437, BOPA2_12_11529, BOPA2_12_30301			*Lr_all*, *Lr_6*, *Lr_6Eur*		
	BOPA1_3568–149			*Lr_all*, *Lr_2Eth*		

**Fig. 4:**
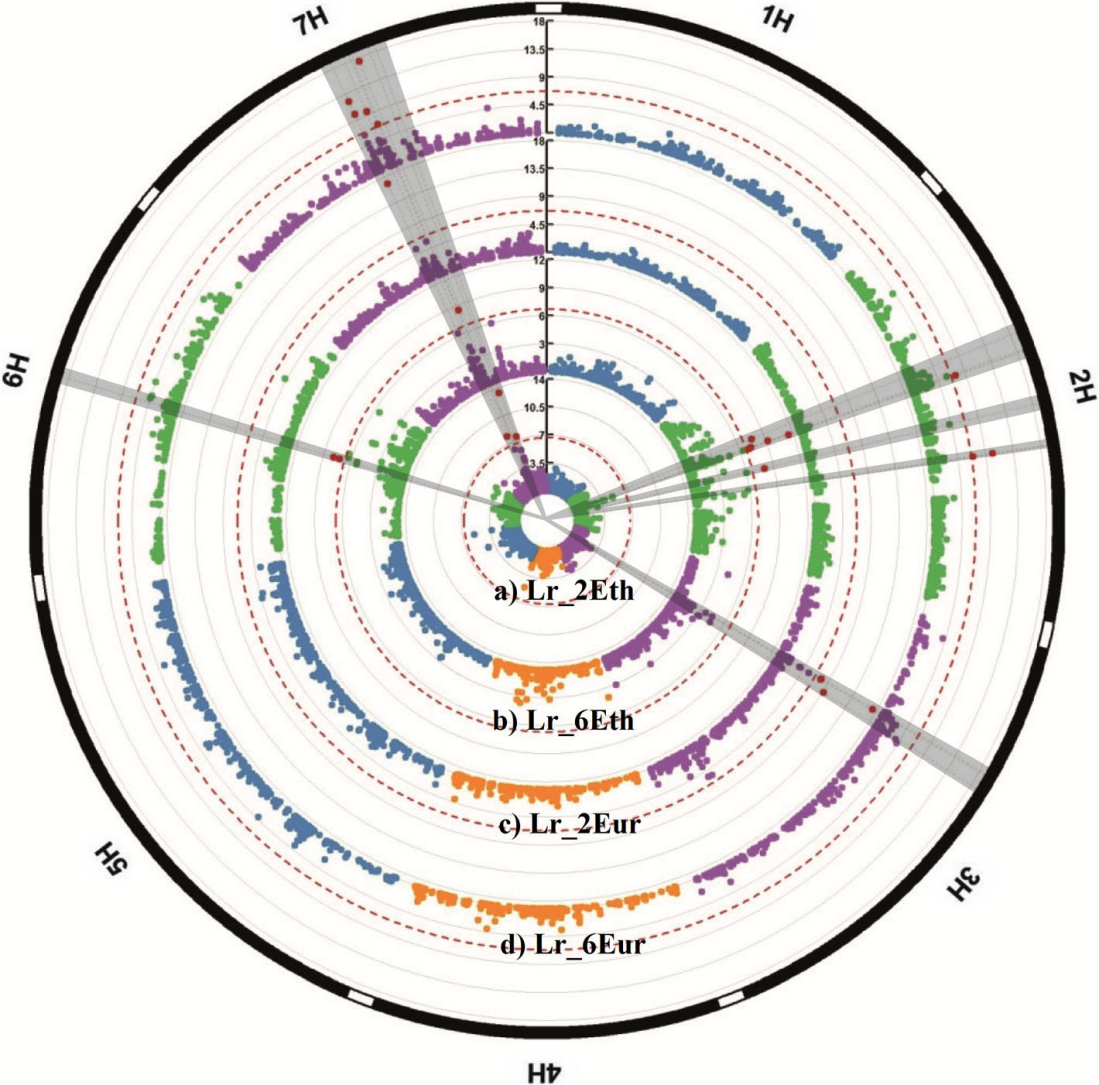
Circular Manhattan plots of GWAS for the seed morphotype in **a**) the 2-rowed panel including naked barleys only from Ethiopia (*Lr_2Eth*), **b**) 6-rowed naked barleys only from Ethiopia (*Lr_6Eth*), **c**) 2-rowed panel including naked barleys only from Europe and Asia (*Lr_2Eur*) and **d**) 6-rowed panel including naked barley only from Europe and Asia (*Lr_6Eur*). The significance level of SNPs (−logP values) is indicated on the vertical scale bar. Individual chromosomes are represented on the outer circle and separated from each other by white borders. Threshold for the FDR (0.05) is indicated by solid circular dash lines. Genomic regions of detected QTL on the respective chromosomes are coloured in red (outer circle). Owing to the limited resolution of the figure single dots can represent multiple SNPs, especially if they have the same or very similar -logP values

In the 2-rowed panel including only naked barleys from Ethiopia *Lr_2Eth*, four SNPs were significantly associated with the occurrence of naked seeds mapping to chromosome 7HL at 70.8 cM (−logP = 8.0), 73.2 cM (−logP = 6.1), 84.4 cM (−logP = 13.4), and 86.6 cM (−logP = 7.5) (Fig. 4). The highest association explained 34% of the total phenotypic variance. Conversely, analysis of the 2-rowed panel *Lr_2Eur* with naked types originating only from Eurasia yielded two loci on 3H and 7HL. The highest association on 3H (46 cM, −logP = 17.4) explained 24.4% and the highest association on 7HL (70.8 cM, −logP = 16) 24% of the total phenotypic variance (Additional file 3: Table S5). The SNPs on 7H at 73.2 cM, 84.4 cM and 86.6 cM that were detected in the entire 2-rowed panel (*Lr_2*) and 2-rowed panel with naked barley from Ethiopia (*Lr_2Eth*) failed the MAF of 5% in *Lr_2Eur*. Regarding subpanel *Lr_6Eth*, four loci were detected on chromosomes 2H, 6H and 7HL (Fig. 4). The highest associations on 2H mapped at 58.8 and 58.9 cM (−logP = 11.5). A second locus on 2H at 76.8 cM had a -logP of 8. On 6H, two SNPs were detected at 65.9 cM (−logP = 7.3 and –logP = 7.9). Only a single significant SNP was detected on 7H at 78.1 cM (−logP = 8.5) (Additional file 3: Table S5).

Analysis of subpanel *Lr_6Eur* revealed associations on 2H and 7HL with a total of ten SNPs (Fig. 4). Six of the SNPs are located on chromosome 7HL delimiting an interval of 9 cM and peaking at 79.8 cM (−logP = 17.5). Four associations were detected on 2H at 58.9 cM (2 SNPs, −logP = 7.3) and 91.2 cM (2 SNPs, −logP = 10.2 and –logP = 7.0).

Chromosomal regions detected in all GWAS panels and subpanels are summarized in Table 1 and all significant SNP detected in each of the panels together with SNP alleles are summarized and presented as supplementary data (Additional files 4 and 5).

### PCR analysis to detect deletion at Nud locus

**Fig. 5:**
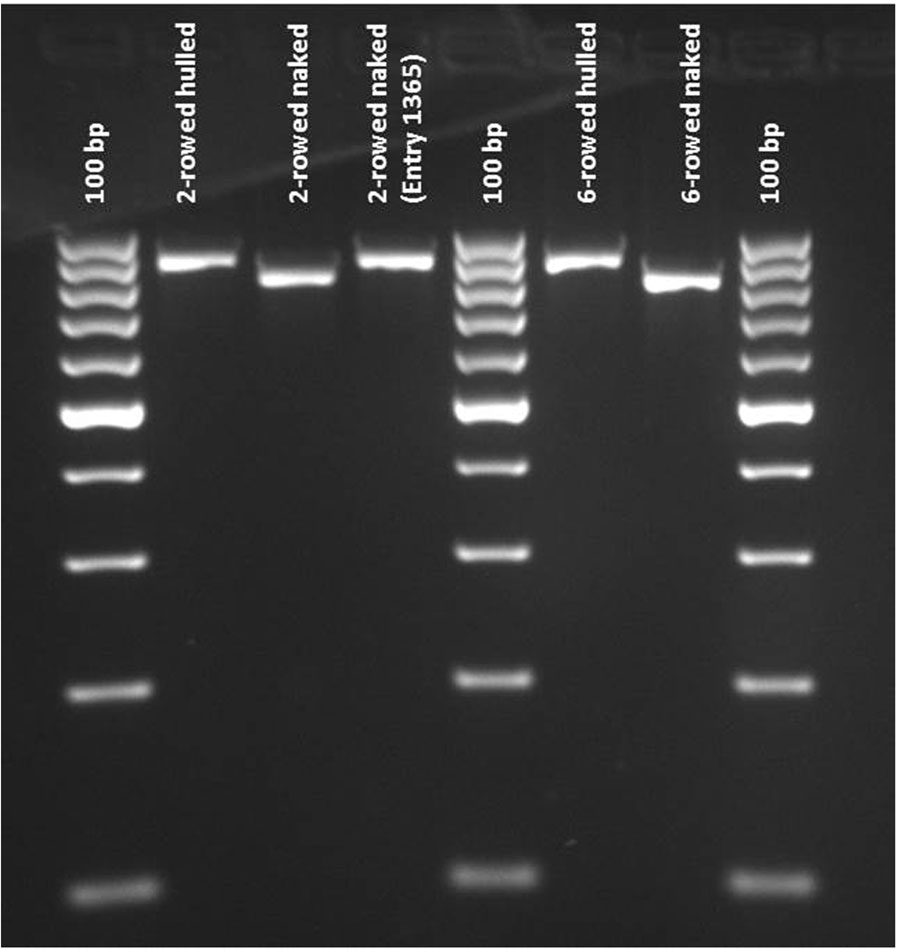
Gel analysis of 2- and 6-rowed accessions. An approximate 900 bp amplicon size was detected for both 2-and 6–rowed hulled accessions as well as for Entry 1365 (naked 2-rowed accession), while all naked barley detected an amplicon size of approximately 800 bp. Size standard (left, right, middle) 100 bp ladder

Taketa [13] have demonstrated that the formation of naked seeds is due to a deletion of about 17 kb on chromosome 7HL encompassing the *Nud* gene corresponding to the region of our main QTL. To examine the presence of this deletion in all naked barley accessions in our panel, the 17 kb region was screened by using a diagnostic PCR assay that is based on a combination of three primers as previously described [13]. According to Taketa [13] hulled and naked accessions should yield product sizes of 853 and 785 bp, respectively. Two hulled accessions of each row type were included as positive controls. All hulled barley accessions yielded an amplification product of approximately 900 bp, while almost all naked accessions yielded a smaller product size of approximately 800 bp. Only a single naked 2-rowed accession (Entry 1365, HOR1145 which originates from Ethiopia) deviated from this pattern giving an amplification product identical to the hulled accessions (Fig. 5). To confirm this unexpected result, additional seeds of Entry 1365 were sown, and further PCR was performed on newly isolated DNA in order to rule out any cross-contamination of DNA. Moreover, the phenotype was verified as naked for all seed sources of this accession. Hence, this genotype, despite being naked, might carry a wild-type allele at the *Nud* locus.

### Confirmation of allelic state at the 17 kb encompassing the Nud

**Fig. 6:**
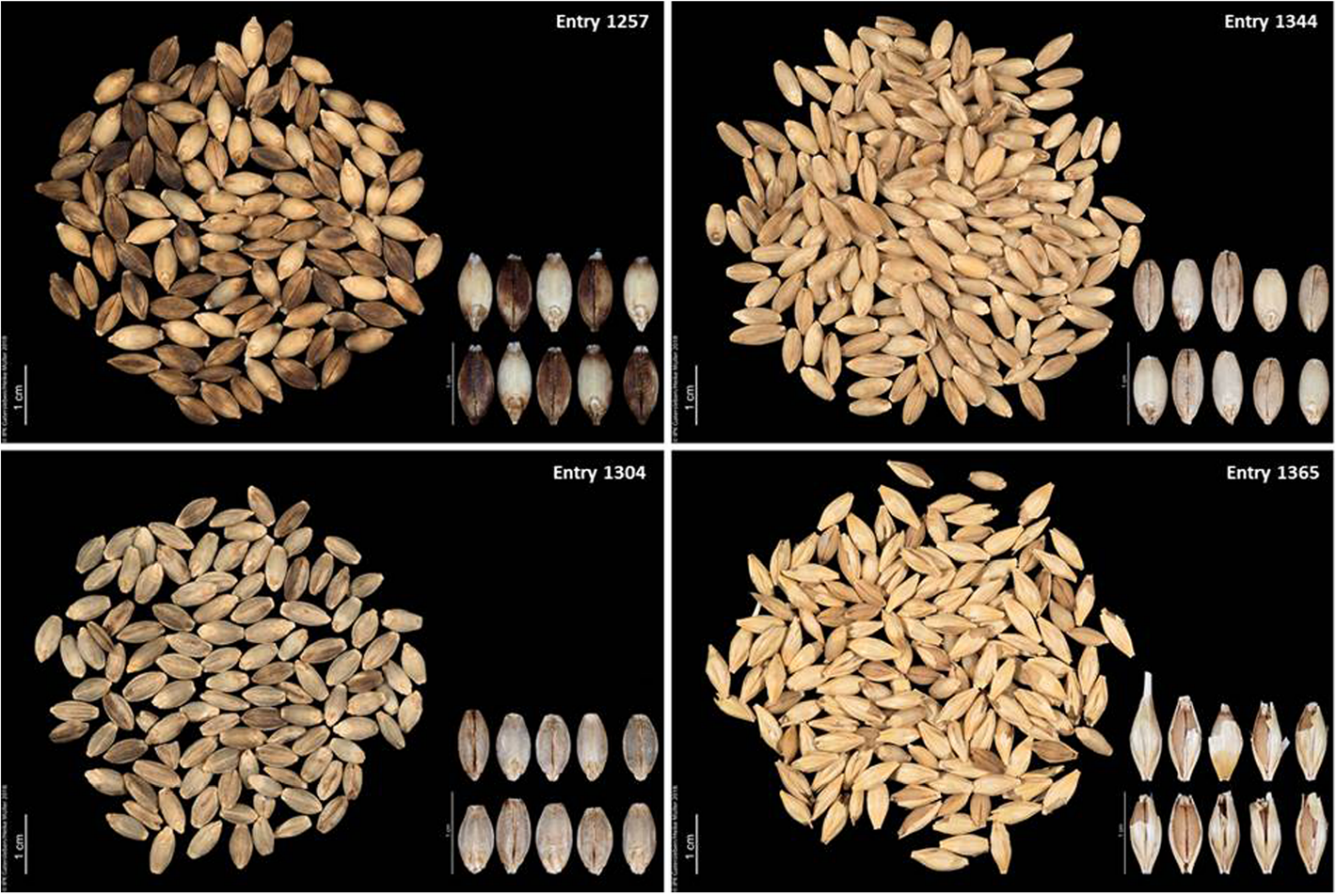
Morphotype of different naked barley accessions after threshing. A marked difference can be seen with Entry 1365 where majority of seeds still remain partially enclosed in the hulls

To further confirm the presence of the 17 kb deletion encompassing the *Nud locus*, PCR fragments of all naked accessions used in the current study were sequenced (primer combinations: wF2, tR2 for covered and wF2, KR1 for naked barley). Sequences of all naked accessions and randomly selected covered accessions (two 2-rowed and two 6-rowed accessions) were aligned to a 17 kb reference sequence derived from cv Morex, which has covered caryopses and carries the intact *Nud* locus. Sequence alignments confirmed that all naked genotypes were characterized by the 17 kb deletion spanning *Nud* except for entry number 1365 that does not show the deletion. After manual threshing, this accession revealed an intermediate phenotype different from the other naked accessions (Fig. 6). Here, the majority of the seeds remained partially enclosed by the hulls. For verification additional seeds from the original genebank stock were checked.

### Seed protein content

Based on the mapping information provided at the “barleyhub” website (barleyhub.org/barley/breeding/) an overlap was detected between the 3H QTL observed in the *Lr_2* and *Lr_2Eur* panel and *Epb1*, a major barley cysteine proteinase involved in endosperm protein degradation. This coincidence motivated us to analyse seed protein content in the present GWAS panel. Compared to naked barleys hulled barleys are generally characterized by a lower seed protein content [35, 36]. By screening the naked accessions of the current study, we investigated the variation in protein content between various subpanels. This could shed some light into the phenotypic significance of the additional QTL detected along with the already reported *Nud*. In the two subpanels of 2- and the 6-rowed accessions, the seed protein content of Ethiopian naked barley was significantly higher compared to the Eurasian naked barleys. However, within Ethiopian barleys, the protein content of 2-rowed and 6-rowed naked accessions did not reveal any significant difference. Similarly, no significant differences were observed between 2-rowed and 6-rowed naked accessions from the Eurasian group. The average protein content for each subpanel is presented as boxplots (Additional file 3: Figure S5a and S5b).

## Discussion

The formation of hulled seeds represents a distinctive characteristic of barley since no other crop of the *Poaceae* family shows this character. To dissect its genetic architecture, we performed GWAS on a panel of spring barley landraces comprising 222 2-rowed and 303 6-rowed accessions. The results revealed that loci on four different chromosomes 2H, 3H, 6H, and 7H were associated with the adherence of husks to the caryopsis. Marker-trait associations for the first three chromosomes turned out to be subpopulation-specific, while associations on 7H were detected across all subpopulations (Table 1).

### Nud is a major locus for the naked caryopsis

The 7H locus was detected in *Lr_all* as well as all subpopulations. Hence, the 7H locus represents the major and geographically most widespread locus influencing the adhesion of hulls to the mature seeds.

The regulation of cutin biosynthesis pathways by *Nud* leads to a highly permeable lipid layer on the caryopsis responsible for hull-caryopsis fusion. The deletion or a low expression level of the *Nud* gene entails a naked caryopsis phenotype [37]. High associations were observed on 7H in a chromosomal region ranging from 70.8 to 86.6 cM. Since *Nud*-specific SNPs are not included on the SNP array employed in this study, the position of the gene was estimated to be at 78.33 cM (MLOC_59305.1) by comparison of the SNP data from this study and to the reference sequence available [38]. This position is in good agreement with the approximate location (75–80 cM) given by [39]. As expected, GWAS within the entire panel revealed the highest association on 7H for marker BOPA2_12_30301, mapping at 79.8 cM, most closely to the putative genetic position of the *Nud* gene at (78.33 cM or 546.6 Mbp, [38]) (Additional file 3: Table S4). The same result was observed for the subpanels *Lr_6*, and *Lr_6Eur*, while the subpanel *Lr_6Eth* peaked at 78.1 cM (BOPA2_12_30996) even closer to the genetic position of the causal gene (Additional file 3: Table S5). Altogether, this supports the hypothesis that in these populations naked seeds are the result of genetic variation in *Nud*. By contrast, the 2-rowed subpanels *Lr_2Eth* and *Lr_2Eur* revealed the highest associations at 84.4 cM and 70.8 cM, respectively.

The wide range of associations flanking the putative locus of the *Nud* gene up to 7.5 and 8.3 cM to both sides reflects the complex pattern of linkage disequilibrium among the corresponding markers (Additional file 3: Figure S6a) corresponding to previous findings of by [34].

### Novel associations identified by analysis of subpopulations

Until now, *Nud* on chromosome 7H is the only cloned gene reported to control the naked barley caryopsis trait, which supports a monophyletic origin of domesticated naked barley [13]. However, other authors have suggested more than one origin of domesticated naked barley [3, 40]. Pasam et al. [21] reported a low level of genetic relatedness (0.49) between naked barley from Ethiopia and other geographical origins and further suggested at least two evolutionary lineages of the naked barley, both of which probably originated from the Eastern Fertile Crescent. In this regard, the detection of additional loci indicates a complex evolutionary history of naked barley.

In addition to the 7H locus, GWAS of *Lr_all* revealed the presence of a second hitherto unknown association on chromosome 2H at 91 cM (Fig. 3, Additional file 3: Table S4). Since spike type is a major determinant of population structure, a separate analysis of the 2- and 6-rowed panels was performed to investigate whether the new locus would be confined to one of the two subpopulations. This approach revealed that the 2H locus is only present in 6-rowed accessions. When separately analyzing naked barley lines from Ethiopia and Eurasia, this QTL could be traced back to *Lr_6Eur*. A second locus on 2H at ~ 58 cM was dected in both six-rowed subpanels *Lr_6Eur* and *Lr_6Eth*. While in *Lr_6Eth* the number of associated SNPs increased spanning a genetic interval from 52.3–58.8 cM, only two SNPs of that locus were significant in *Lr_6Eur*. The panel of *Lr_6Eth* revealed a third locus on 2H at 76.8 cM (Table 1). The highest associations in the Ethiopian subpanel were found on 2H at about 58–59 cM in the centromeric region containing the flowering time gene *HvCEN*. The extent of LD between associated SNPs on 2H shows a complex pattern indicating the possibility that this region might harbor several loci and not only one (Additional file 3: Figure S6b). The co-localization with a flowering time gene might arise from the fact that more hulled barley accessions originate from Europe, while the majority of naked accessions comes from the region West Asia and North Africa (WANA). Therefore, the QTL in the vicinity of *HvCEN* might arise from the underlying population stratification regarding European origin (hulled) vs WANA (majority of naked), while the role of the other two QTL on 2HL remains unclear.

Similarly, analysis of *Lr_2* yielded a novel locus on chromosome 3H (Fig. 3). Further subdivision of the naked barley lines in *Lr_2Eth* and *Lr2_Eur* demonstrated that the association is confined to naked accessions from Eurasia (Fig. 4). LD analysis of all significant SNPs detected on 3H in *Lr_2* revealed a high level of LD between the four markers at positions 45.4 and 46 cM, while the SNP at position 51.1 cM shows only moderate LD to the other SNPs (Additional file 3: Figure S6c). However, when only Eurasian naked barleys were considered in the analysis (*Lr_2Eur*), all 3H SNPs were in high LD with each other and with the *Nud* locus (Additional file 3: Figure S6d).

Finally, one association on 6H was detected only in 6-rowed accessions from Ethiopia (Fig. 4, Additional file 3: Table S5). It is marked by two SNPs at 65.9 cM, which are in very high LD (Additional file 3: Figure S6e).

In a recent association analysis of a large germplasm set comprising 2417 accessions fingerprinted with the same iSelect array, significant associations were detected with SNPs located around the *Nud* locus as well as with unmapped SNPs [34]which might correspond to additional loci as in our study. However, inspection of the updated positions of these unmapped SNPs revealed their location on 7H, close to the *Nud* locus. A possible reason why no footprints of selection were detected in this study is the low frequency of naked accessions in the corresponding panel (9% vs 17% in the present study). The analysis of subpopulations, as it was performed in the present study, proved crucial for the detection of these loci, likely due to the increase in allele frequencies and the reduction of confounding effects. The detection of multiple, subpopulation-specific associations might be the result of two different scenarios. In the first scenario, this reflects a complex genetic architecture of the naked caryopsis trait, giving rise to several QTL involved in trait expression. Alternatively, the observed QTL represent footprints of the selection of additional traits in naked barley following a geographic (Ethiopia vs Eurasia) or morphological pattern (2-rowed vs 6-rowed).

### PCR analysis reveals Nud as the main locus for hull-caryopsis adherence

Regarding the first scenario, PCR results and sequence data revealed, that *Nud* is the main locus involved in hull-caryopsis adherence. This is supported by our GWAS results and confirms the hypothesis of [13] that *Nud* is completely deleted in all naked accessions. Nevertheless, one landrace originating from Ethiopia (Entry 1365) yielded an amplicon of the diagnostic size for covered barley. This is an indication that, although this accession is hull-less, the entire 17 kb fragment is not deleted as would have been expected. Also, the phenotype of this accession showed increased hull-caryopsis adherence (Fig. 6). Whether this is due to the presence of a second locus other than *Nud* or owed to a different mutation of *Nud,* not resulting in a size polymorphism, needs to be further investigated. The primer pair, wF2, and tR2, which amplified an approximately 850 bp fragment in covered barley, binds at a far distance (around 2400 bp) off the genetic position of the *Nud* gene. Hence, the PCR assay does not reflect the allelic status of the gene itself. On the other hand, the presence of additional allelic variation at the *Nud* locus or even a mutation in another gene giving rise to naked caryopses would be anything but unexpected, given the large number of genes involved in the biosynthesis of lipids [41] and the presumption that there could be a quantitative variation in the formation of the cuticular lipid covering the pericarp hence making the naked trait a quantitative rather than a qualitative trait [34].

### Novel associations indicate footprints of selection

Munoz-Amatriain et al. [34] predicted the presence of additional QTL other than *Nud* as a result of a difference in the degree of hull adhesion and suggested the presence/absence of husks be a quantitative rather than a qualitative trait.

As we could not see any phenotypic differences between naked accessions except one accession (entry 1365) and the deletion at the *Nud* locus is pervasive in naked barley except for entry 1365, it is deemed unlikely, that the associations detected outside the *Nud* locus are functionally related to the formation of a naked caryopsis. Thus, we suspect that the observed associations on chromosomes 2H, 3H, 6H instead represent footprints of selection for hitherto unknown adaptive or end-use traits. Since, seed protein content is a well-known determinant of the nutritional value of seeds, we investigated, if the presence of the identified footprints of selection was correlated with seed protein content.

Compared to hulled barley, naked barley has an increased protein content [35, 36]. Also, this study revealed differences in protein content between naked accessions of different origin (Eurasian having a lower protein content than Ethiopian landraces).

The QTL region on 3H (45.4–51.1 cM) detected in the 2-rowed Eurasian panel harbors *Epb1* (http://www.barleyhub.org/barley/breeding/), a major barley cysteine proteinase involved in endosperm protein degradation [42]. The difference in protein content between naked barleys from Eurasia and Ethiopia could a result of unconscious or directed selection towards different end-uses, especially, as naked barley is one of the primary food sources in Ethiopia. However, since the 3H QTL was restricted to the *Lr_2Eur* subpanel, it seems unlikely, that allelic variation in Epb1 would account for the observed differences in protein content of 2-rowed and 6-rowed naked barleys from the Ethiopian and Eurasian subgroups. Moreover, inspection of the recently published reference sequence of barley revealed two paralogues, *Epb1* and *Epb2* (GB accession no. U19359.1 and U19384.1, respectively) which are very similar (94% sequence identity by comparing both sequences). By performing a BLAST analysis of both genes against genomes of different barley lines (three cultivars, a landrace and a wild barley line, M. Mascher personal communication), we found that both genes are mapping outside the QTL region 109 cM or 636 Mbp, spaced at a distance of only 20 kb.

## Conclusion

The results of the present study demonstrate that the naked phenotype in spring barley is based on the deletion on chromosome 7H of a 17 kb region carrying the *Nud* gene. However, one of the naked landraces from Ethiopia lacking the deletion showed an intermediate phenotype with slightly adhering hulls. Therefore, the phenotype of this accession could be either caused by the presence of a mutation affecting the function of *Nud,* and that is not detected by the PCR assay (e.g. a smaller deletion, or a point mutation). Alternatively, the phenotype is caused by genetic variation in genes other than *Nud*. Moreover, we identified characteristic footprints of selection for naked caryopsis in different geographic areas. These may have arisen from different end-use preferences or reflect selection for adaptive traits yet to be discovered.

## Additional files

Additional file 1: Panel of 222 two-rowed spring barley landraces (accession name, origin, geo-reference information, botanical nomenclature, row type and caryposis type). (XLSX 25 kb)

Additional file 2: Panel of 303 six-rowed spring barley landraces (accession name, origin, geo-reference information, botanical nomenclature, row type and caryposis type). (XLSX 30 kb)

Additional file 3: Supporting information on methods and results (PCR conditions and primers, origin and distribution of landraces, average LD decay, information on associated SNPs in all subpanels, locus-specific LD plots). (DOCX 5841 kb)

Additional file 4: Allele distribution (significantly associated SNPs from all panels within two-rowed landraces). (XLSX 28 kb)

Additional file 5: Allele distribution (significantly associated SNPs from all panels within six-rowed landraces). (XLSX 44 kb)

## Abbreviations

FDR: False discovery rate
GWAS: Genome-wide association study
LD: Linkage disequilibrium decay
MAF: Minor allele frequency
PCA: Principal component analysis
QTL: Quantitative trait locus
SNP: Single nucleotide polymorphism
SSR: Single sequence repeat
