# Supplementary material for: A tiered approach to genome-wide association analysis for the adherence of hulls to the caryopsis of barley seeds reveals footprints of selection
Source: BMC Plant Biol. 2019 Mar 6;19:95. doi: 10.1186/s12870-019-1694-1 (PMC6404267; doi:10.1186/s12870-019-1694-1)
Supplement: Supplementary file 3 — Supporting information on methods and results (PCR conditions and primers, origin and distribution of landraces, average LD decay, information on associated SNPs in all subpanels, locus-specific LD plots). (DOCX 5841 kb) [file 12870_2019_1694_MOESM3_ESM.docx]

**A tiered approach to genome-wide association analysis for the adherence of hulls to the caryopsis of barley seeds reveals footprints of selection**

Celestine Wabila^1^, Kerstin Neumann^1^, Benjamin Kilian^1,2^, Volodymyr Radchuk^1^, *Andreas Graner^1^

^1^Leibniz Institute of Plant Genetics and Crop Plant Research (IPK), 06466 Gatersleben, Germany

^2^Current address: Global Crop Diversity Trust, Platz der Vereinten Nationen 7, 53113 Bonn, Germany

*Corresponding author

**Supplementary data**

**Table S3………………………………………………………………………………………2**

**Table S4………………………………………………………………………………………2**

**Table S5………………………………………………………………………………………3**

**Figure S1……………………………………………………………………………………..5**

**Figure S2……………………………………………………………………………………..6**

**Figure S3……………………………………………………………………………………..7**

**Figure S4……………………………………………………………………………………..7**

**Figure S5a……………………………………………………………………………………8**

**Figure S5b……………………………………………………………………………………8**

**Figure S6………………………………………………………………………………….9-11**

Table S3. PCR conditions and primers used in PCR and re-sequencing of the 17 kb region harbouring the *Nud.*

| Master mix | | | PCR profile | | | |
| --- | --- | --- | --- | --- | --- | --- |
| Mix | µl/sample | | 95 °C | 15 min |  |  |
| Water | 11.9 | | 94 °C | 30 sec |  |  |
| 10xPCR Buffer | 2 | | 65°C | 30 sec | 5 cycles | td -1C per cycle |
| dNTPs (2mM) | 2 | | 72 °C | 1 min |  |  |
| Primer F (5µM) | 1 | | 94 °C | 30 sec |  |  |
| Primer R(5µM) | 1 | | 60°C | 30 sec | 35 cycles |  |
| Hot star taq | 0.1 | | 72 °C | 1 min |  |  |
| D N A (20ng/µl) | 2 | | 72 °C | 7 min |  |  |
| Total | 20 | | 8 °C |  | PCR-cycler: | 10 |
|  |  |  |  |  |  |  |
|  | Primers | | | | Expected product size (bp) | Detected product size (bp) |
| Covered | wF2:GCTTGCAGTTACAGAGCTACTACTAC | | | | 835 | 920 |
|  | **tR2:GCGGTCCTTTCTTTCCAGT** | | | |  |  |
| Naked | wF2:GCTTGCAGTTACAGAGCTACTACTAC | | | | 785 | 835 |
|  | **kR1:CCTCACCACTTAACCATGTCTG** | | | |  |  |

Table S4. Top SNPs highly associated with the barley caryopsis type in combined analysis and separate analysis of 2- and 6-rowed GWAS panels.

| **Combined analysis of both 2-rowed and 6-rowed accessions (*Lr_all*), 525 lines** | | | | |
| --- | --- | --- | --- | --- |
| SNPs | Chromosome | Position | -logP | R^2^ |
| SCRI_RS_140972 | 2H | 91.2 | 7.7 | 10.2 |
| BOPA2_12_20685 | 7H | 70.8 | 12.5 | 12 |
| BOPA2_12_11437 | 7H | 70.8 | 10 | 11.5 |
| BOPA1_1676-557 | 7H | 70.8 | 7.9 | 11 |
| BOPA2_12_11529 | 7H | 75.2 | 7 | 9.4 |
| SCRI_RS_124478 | 7H | 76.7 | 6.2 | 9 |
| BOPA2_12_30301 | 7H | 79.8 | 16.4 | 13 |
| SCRI_RS_4562 | 7H | 84.4 | 11 | 12 |
| **2-rowed panel (*Lr_2*), 222 lines** | | | | |
| SNPs | Chromosome | Position | -logP | R^2^ |
| BOPA2_12_10532 | 3H | 45.4 | 7.2 | 15 |
| BOPA1_4256-833 | 3H | 46.0 | 15.1 | 17 |
| BOPA1_2391-566 | 3H | 46.0 | 15.1 | 17 |
| BOPA2_12_30474 | 3H | 46.0 | 7.2 | 14 |
| BOPA2_12_20685 | 7H | 70.8 | 10.3 | 16 |
| BOPA2_12_30301 | 7H | 79.8 | 7.1 | 16 |
| SCRI_RS_4562 | 7H | 84.4 | 15.6 | 21 |
| BOPA1_3568-149 | 7H | 86.6 | 12.1 | 19.8 |
| **6-rowed panel (*Lr_6*), 303 lines** | | | | |
| SNP | Chromosome | Position | -logP | R^2^ (%) |
| SCRI_RS_140972 | 2H | 91.2 | 11 | 14.7 |
| BOPA2_12_20685 | 7H | 70.8 | 8.3 | 13.6 |
| BOPA2_12_11437 | 7H | 70.8 | 7.6 | 13 |
| BOPA1_1676-557 | 7H | 70.8 | 7 | 12.2 |
| BOPA2_12_11529 | 7H | 75.2 | 7.6 | 13 |
| BOPA2_12_30301 | 7H | 79.8 | 11.5 | 14.9 |
| SCRI_RS_4562 | 7H | 84.4 | 5.6 | 12 |

**Table S5.** SNP markers highly associated with the barley caryopsis type in 2-rowed and 6-rowed panels based on geographic origin of naked barleys.

| **2-rowed panel with only Ethiopian naked barley 2-rowed panel (*Lr_2Eth*), 206 lines** | | | | |
| --- | --- | --- | --- | --- |
| SNP | Chromosome | Position | -logP | R^2^(%) |
| BOPA2_12_20685 | 7H | 70.8 | 8 | 30 |
| BOPA2_12_30998 | 7H | 73.2 | 6.1 | 27 |
| SCRI_RS_4562 | 7H | 84.4 | 13.4 | 34 |
| BOPA1_3568-149 | 7H | 86.6 | 7.5 | 31 |
| **2-rowed panel with only Eurasian naked barleys 2-rowed panel (*Lr_2Eur*), 194 lines** | | | | |
| SNP | Chromosome | Position | -logP | R^2^ (%) |
| BOPA2_12_10532 | 3H | 45.4 | 7.7 | 18 |
| BOPA1_4256-833 | 3H | 46 | 17.4 | 24.4 |
| BOPA1_2391-566 | 3H | 46 | 17.4 | 24.4 |
| BOPA2_12_30474 | 3H | 46 | 7.7 | 18 |
| BOPA2_12_30467 | 3H | 51.1 | 9.2 | 19 |
| BOPA2_12_20685 | 7H | 70.8 | 16.8 | 24 |
| **6-rowed panel with only Ethiopian naked barley (*Lr_6Eth*), 285 lines** | | | | |
| SNP | Chromosome | Position | -logP | R^2^ (%) |
| BOPA2_12_11096 | 2H | 52.3 | 7.6 | 13 |
| BOPA1_3355-605 | 2H | 58.1 | 9.2 | 14.2 |
| SCRI_RS_127347 | 2H | 58.1 | 7.3 | 13 |
| BOPA2_12_21476 | 2H | 58.8 | 11.5 | 15.5 |
| BOPA2_12_11316 | 2H | 58.9 | 11.5 | 15.5 |
| BOPA1_4629-162 | 2H | 76.8 | 8 | 13 |
| SCRI_RS_133948 | 6H | 65.9 | 7.3 | 13 |
| SCRI_RS_3016 | 6H | 65.9 | 7.9 | 13 |
| BOPA2_12_30996 | 7H | 78.1 | 8.5 | 14 |
| **6-rowed panel with only Eurasia naked barley (*Lr_6Eur*), 275 lines** | | | | |
| SNP | Chromosome | Position | -logP | R^2^ (%) |
| BOPA2_12_21476 | 2H | 58.8 | 7.4 | 13 |
| BOPA2_12_11316 | 2H | 58.9 | 7.4 | 13 |
| BOPA2_12_11285 | 2H | 91 | 10.2 | 15 |
| SCRI_RS_140972 | 2H | 91.2 | 7 | 13 |
| BOPA2_12_20685 | 7H | 70.8 | 12.2 | 16 |
| BOPA1_1676-557 | 7H | 70.8 | 9.9 | 15 |
| BOPA2_12_11437 | 7H | 70.8 | 6.2 | 12.7 |
| BOPA2_12_11529 | 7H | 75.2 | 9.5 | 14 |
| BOPA2_12_30999 | 7H | 77.3 | 7 | 13 |
| BOPA2_12_30301 | 7H | 79.8 | 17.5 | 17.8 |


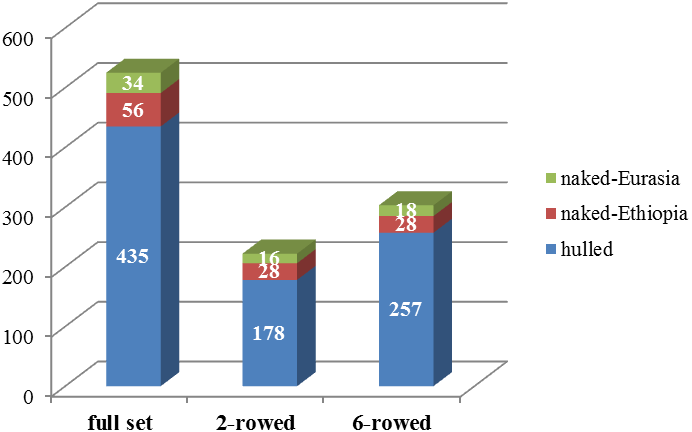


**GWAS panels**

N=525

N=222

N=303

**Number of accessions**

**Figure S1.** Composition of the GWAS panel and the subpanels of 2- and 6-rowed accessions.


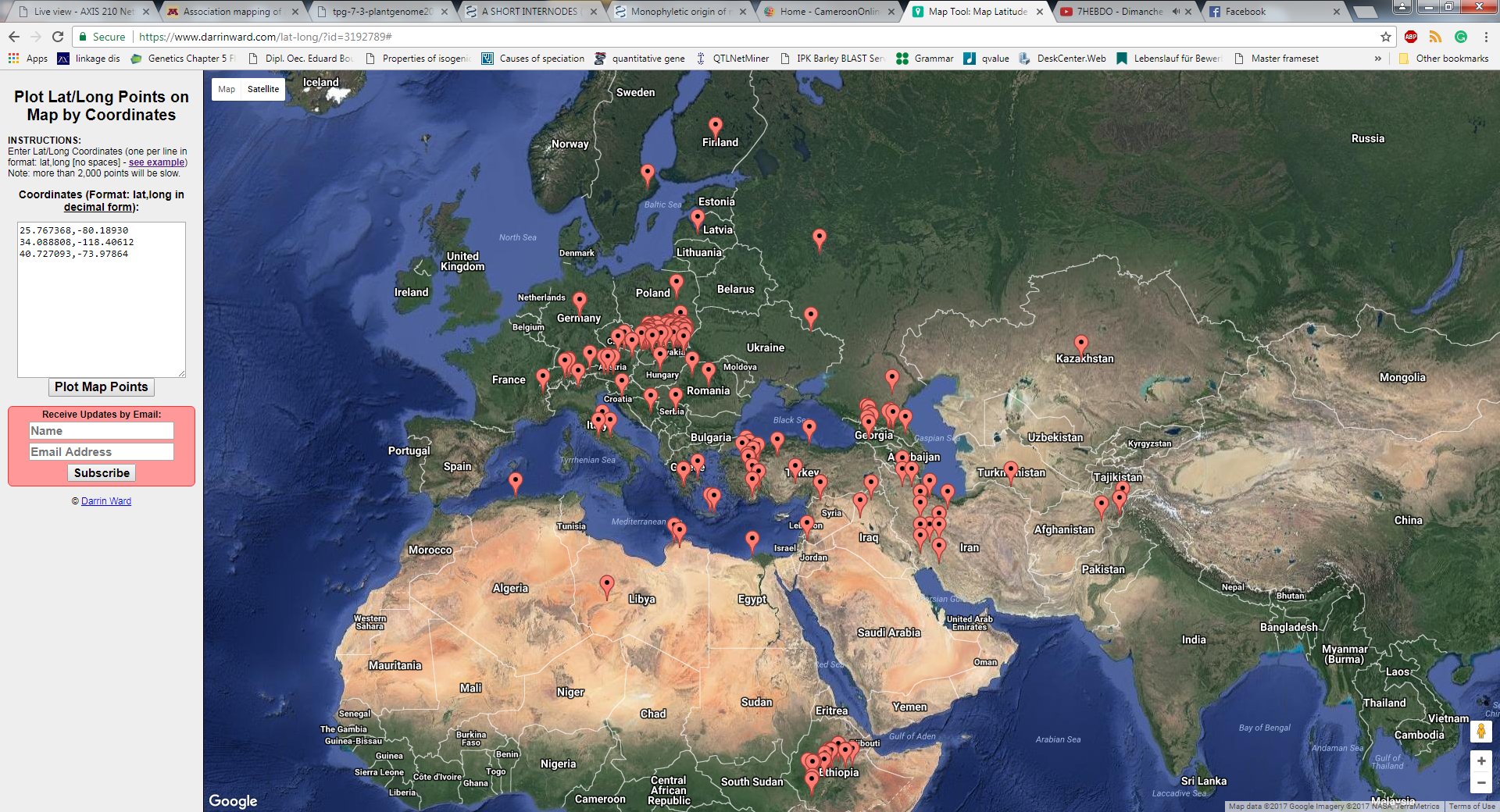


a)

**
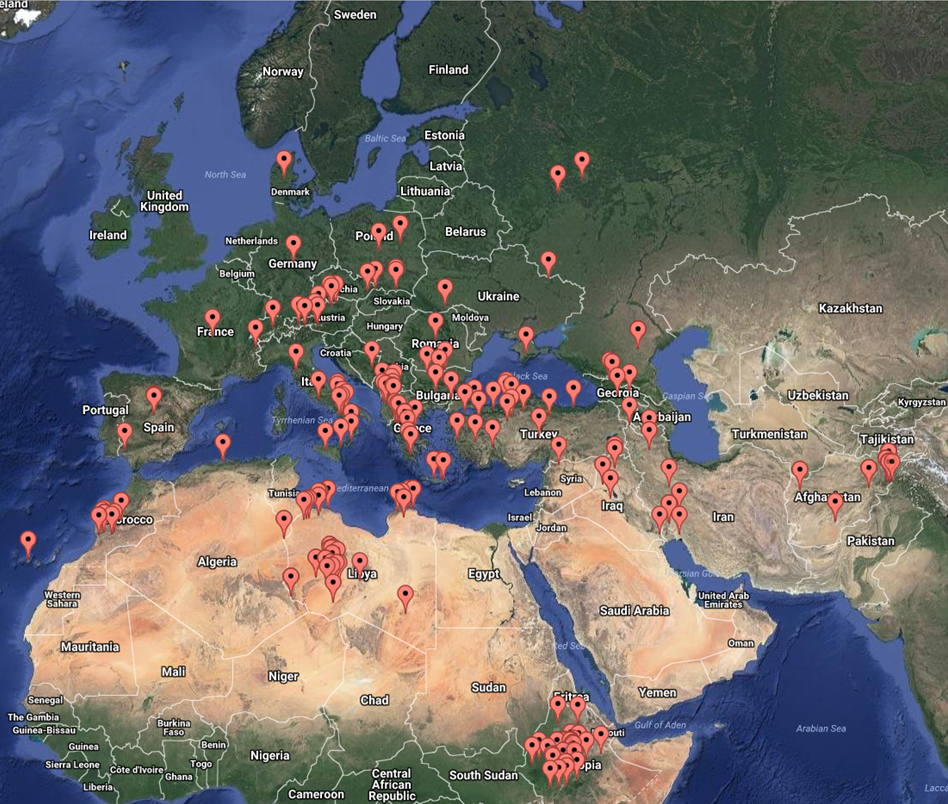
**

b)

**
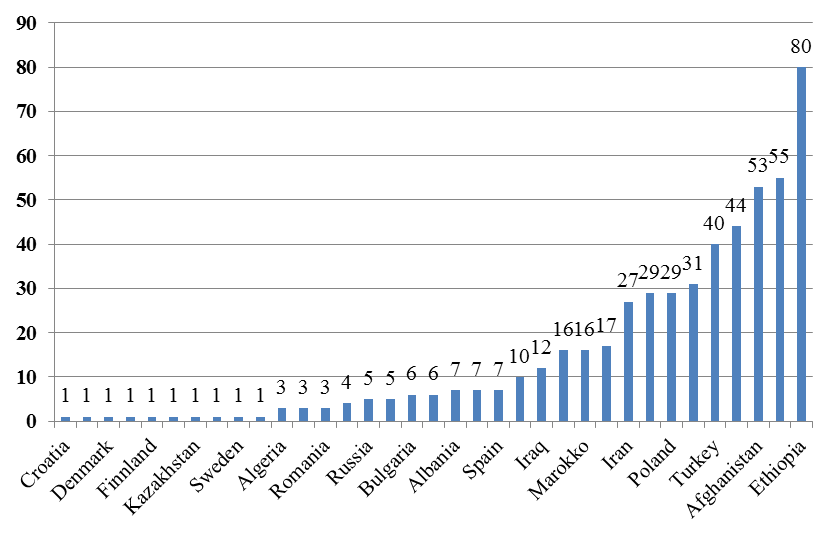
**

C)

**Figure S2.** Geographical distribution of 2-rowed (a) and 6-rowed (b) spring barley accessions. Collection sites are based on latitudes and longitudes. The number of accessions per country is given in panel c.


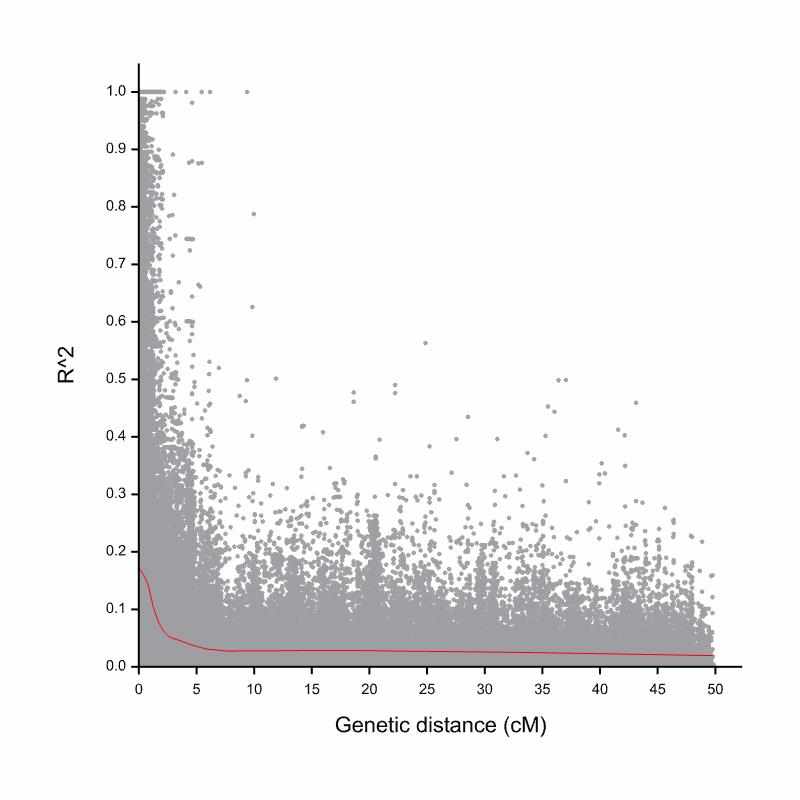

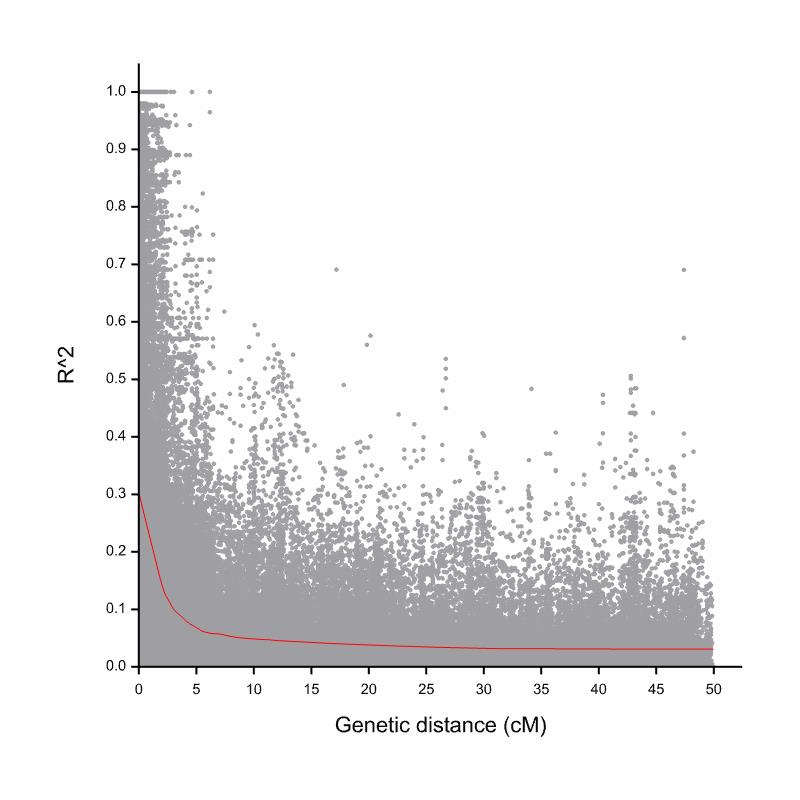


**Figure S3.** Average genome-wide LD decay (*r^2^*) within the 2-rowed panel (left) and the 6-rowed panel (right) for all chromosomes as a function of genetic distance (cM). The dashed line indicates the 95^th^ percentile of the distribution of *r^2^* from all unlinked loci. The Loess fitting curve (red) illustrates the LD decay.


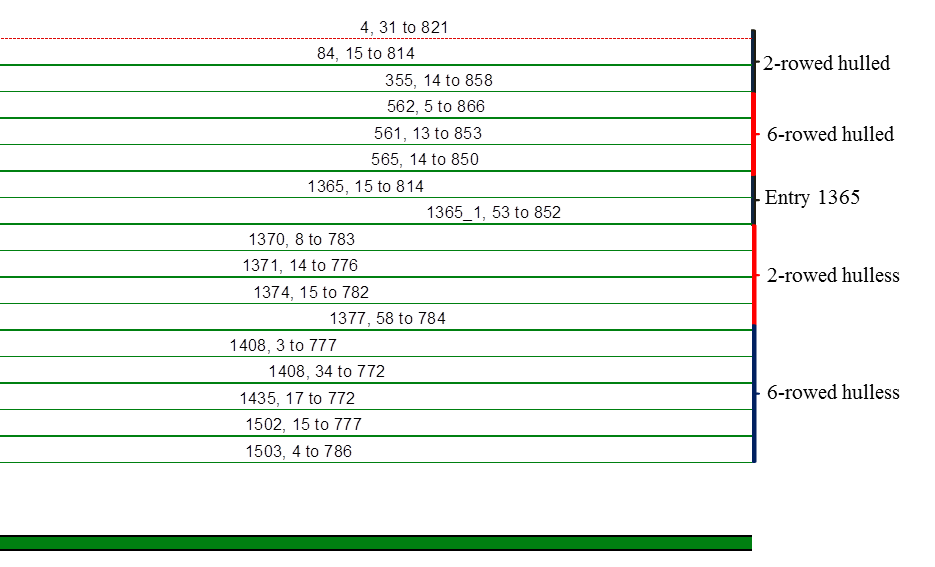
**Figure S4.** Sequence alignment of covered and naked accessions after trimming. Only part of the sequences are shown. Numbers on each sequence starts with the Entry number of the accession followed by start and end of the sequence. Accession 1365 was sown and new DNA isolated to be sure of no DNA contamination, hence this accession is represented twice on the figure.


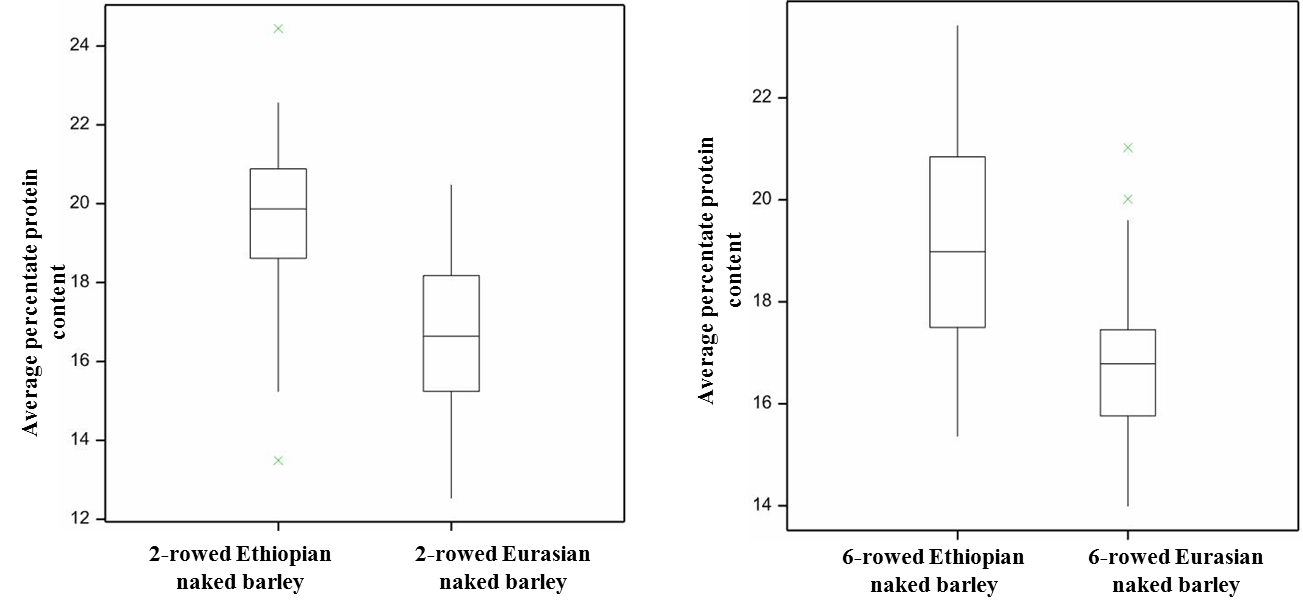


**Figure S5a.** Average percentage protein content between Ethiopian naked barley and Eurasian naked barley in different subpanels (2-rowed and 6-rowed). In both panels a significant difference was observed (t-test P>0.0001)


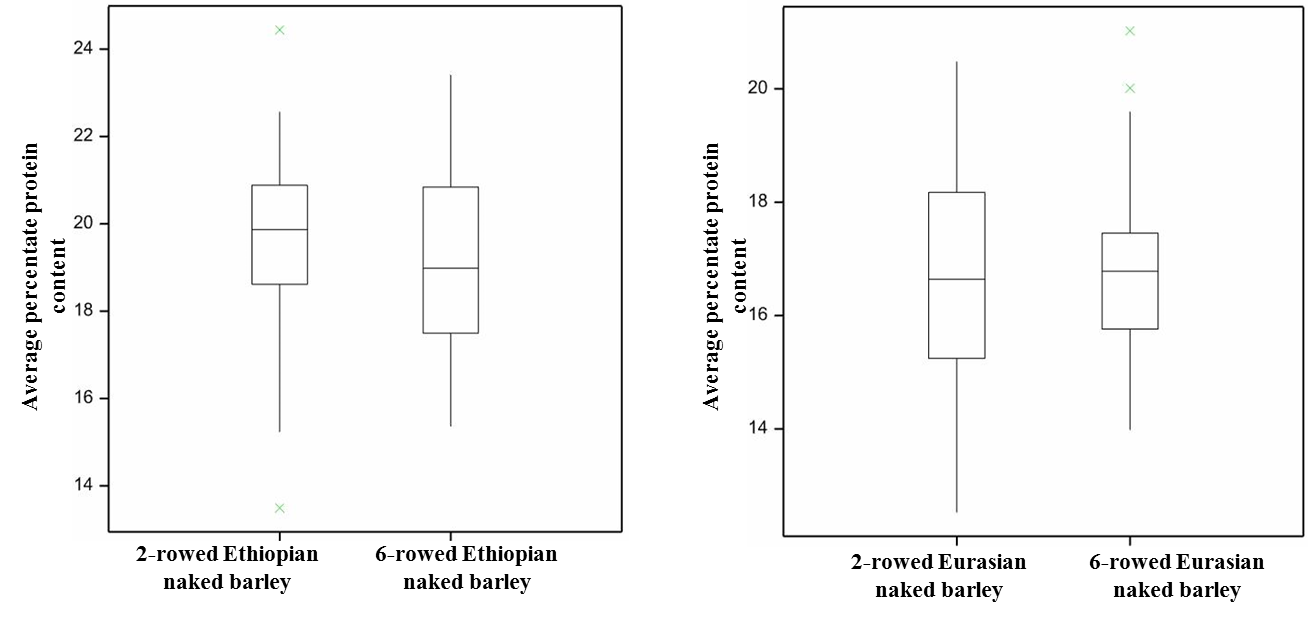


**Figure S5b.** Average percentage protein content between Ethiopian and Eurasian naked barley across both 2- and 6-rowed subpanels. Across both panels, no significant difference was observed between row type subgroups.


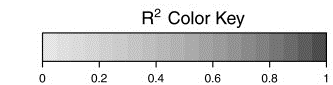

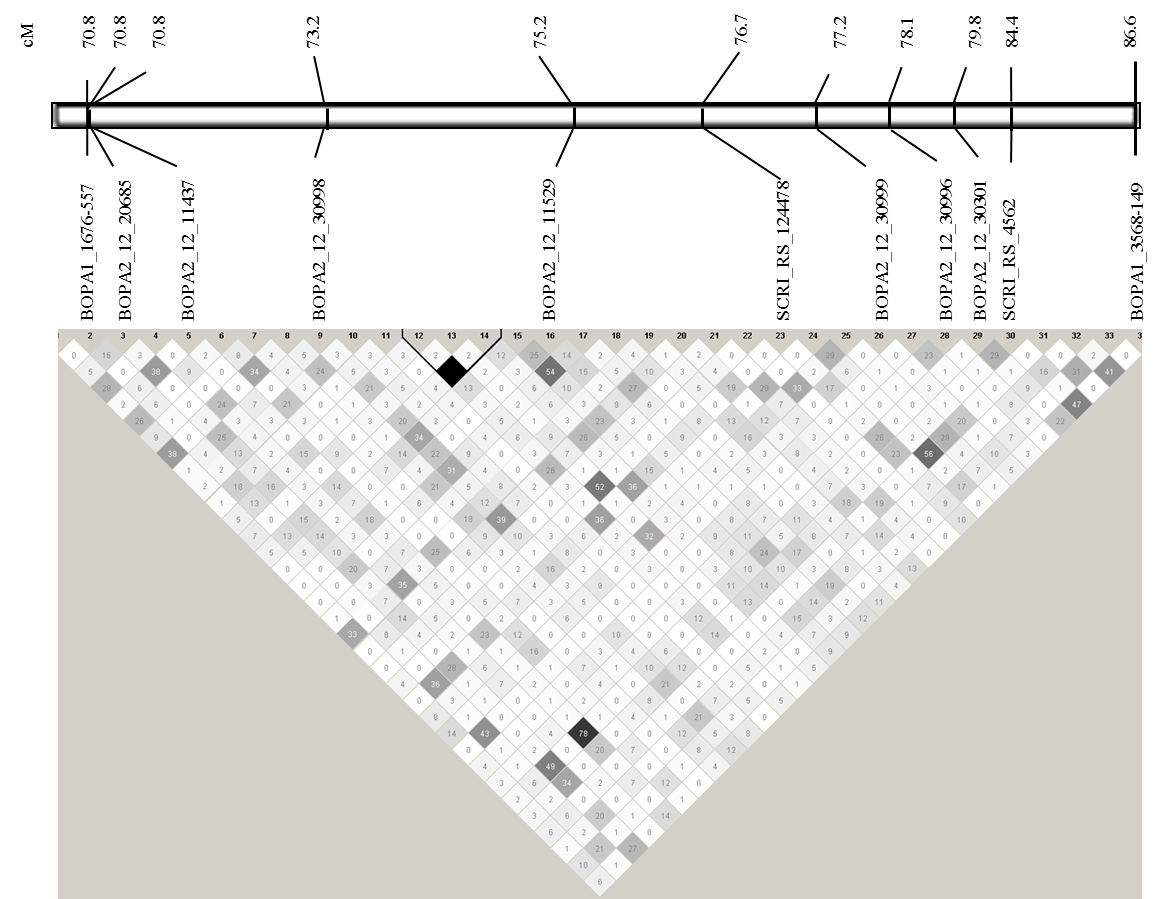


7H *(Lr_all)*

**Figure S6a:**


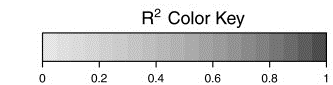

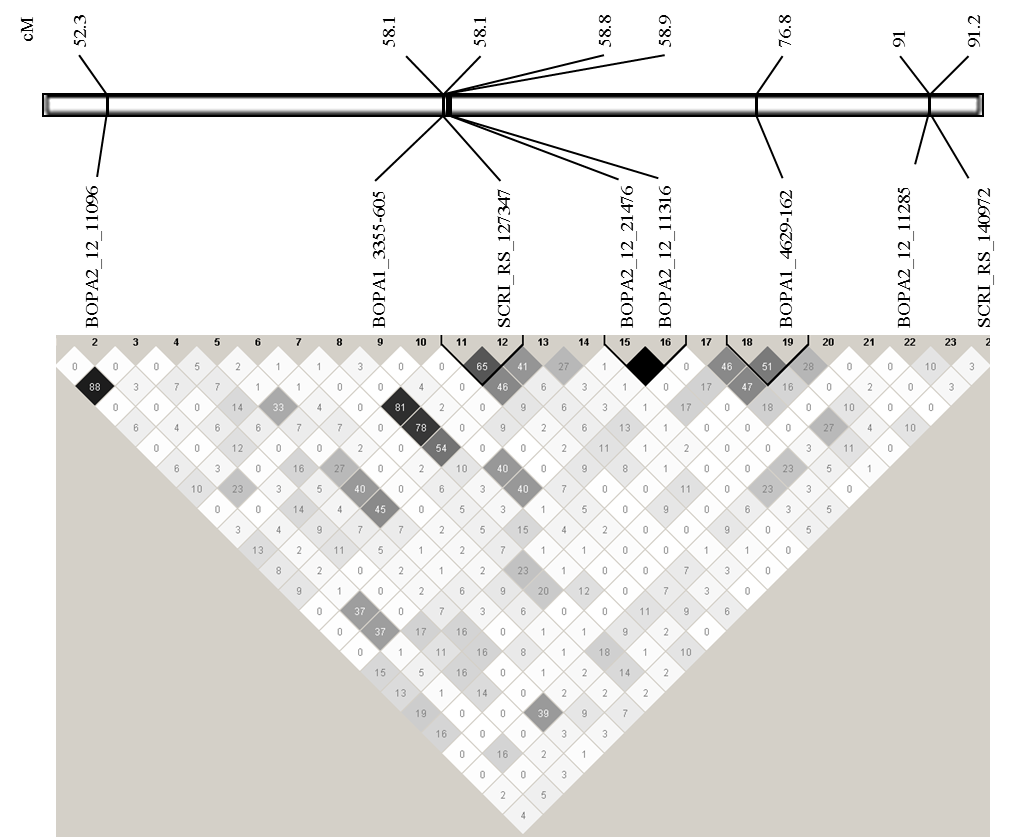


2H (*Lr_all*)

**Figure S6b:**


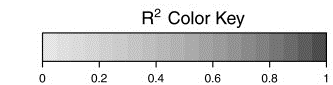

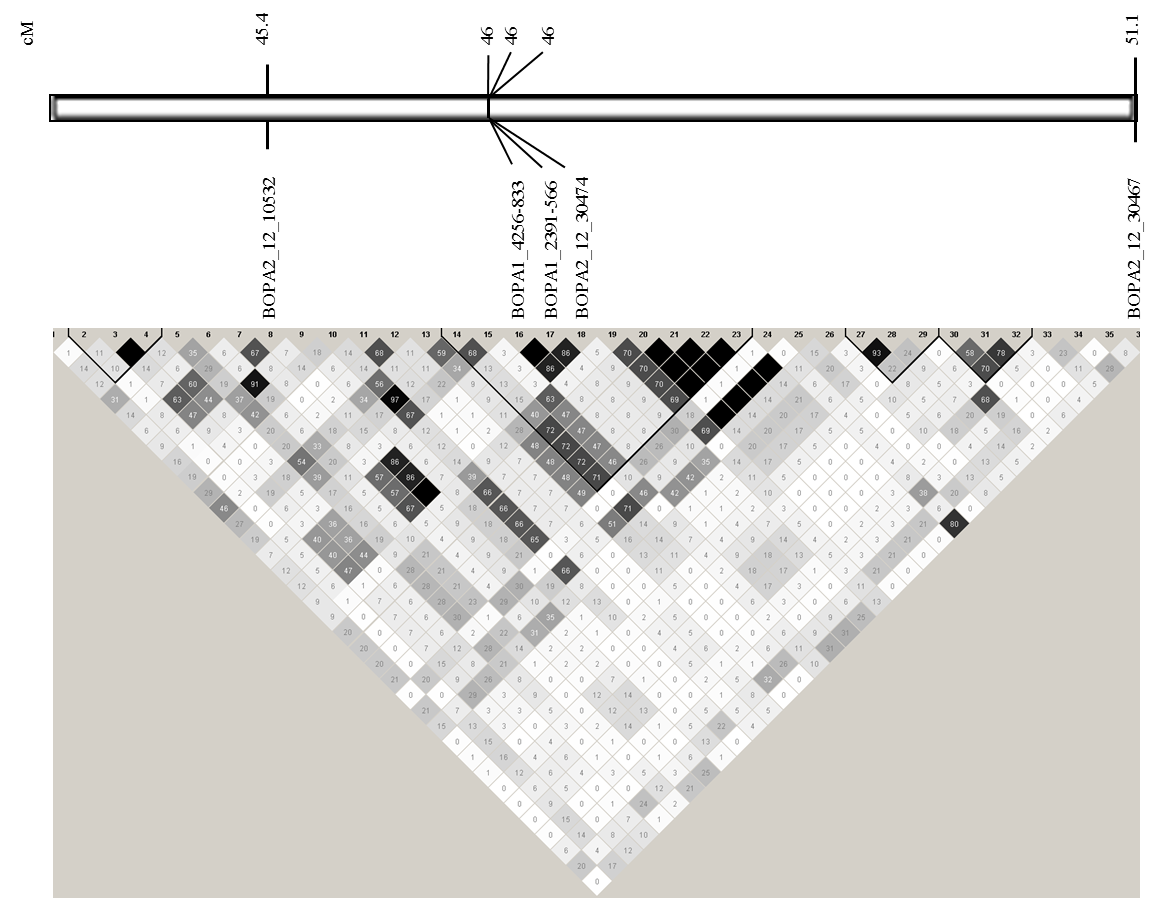


3H (*Lr_all*)

**Figure S6c:**


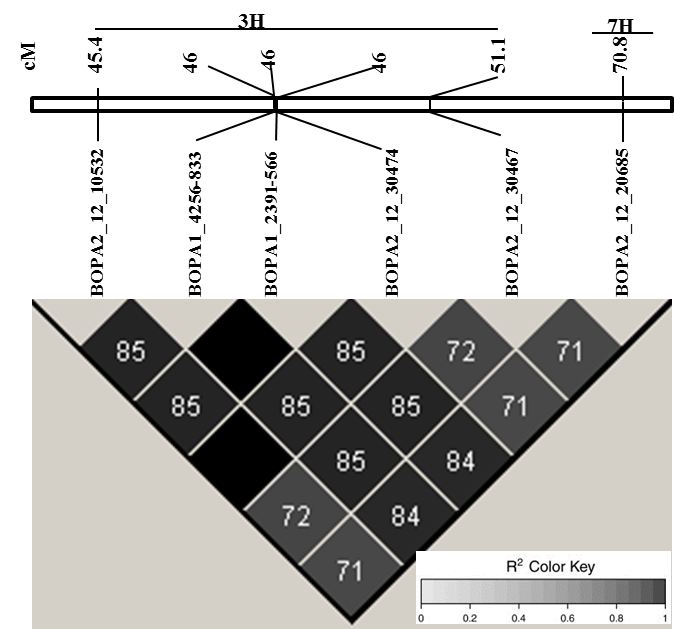


3H vs 7H (*Lr_2Eur*)

**Figure S6d:**


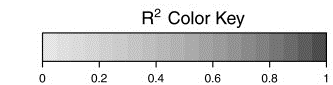

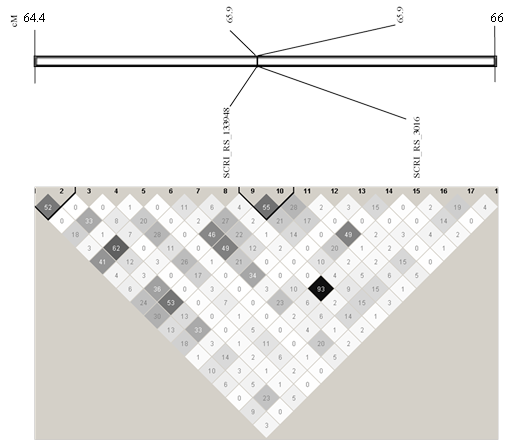


6H (*Lr_all*)

**Figure S6e:**

**Figure S6.** Heat plots showing pair-wise linkage disequilibrium (R^2^) for all SNPs significantly associated on each chromosome. Heat plots were constructed between SNPs detected on individual chromosomes; 7H (a), 2H (b), 3H (c), 3H vs 7H (d), and 6H (e). SNP markers within genomic region of associated SNPs are indicated by numbers and ordered corresponding to their genetic positions. Identifiers of significantly associated SNPs exceeding the FDR threshold are given on top of their respective R^2^ values (in %) along with their genetic positions. Co-segregating SNPs are marked with black borders according to [45]. Only significantly associated SNPs are indicated on each plot. All figures refer to LD in *Lr_all* exepct Fig. 6d who refers to LD within subpanel *Lr_2Eur*.
